# 1249. Stealth Stewardship: Compliance with Antimicrobial Restriction Processes at an Academic Medical Center

**DOI:** 10.1093/ofid/ofad500.1089

**Published:** 2023-11-27

**Authors:** Jihye Kim, Adam Greenfield, Erin N Deja, Kimberly B Lee, Barry Rittmann, Gonzalo Bearman, Sangeeta Sastry

**Affiliations:** VCU Health System, Richmond, Virginia; Virginia Commonwealth University Health System, Henrico, Virginia; Cone Health, Richmond, Virginia; Virginia Commonwealth University Health System, Henrico, Virginia; Virginia Commonwealth University Health System, Henrico, Virginia; Virginia Commonwealth University Health System, Henrico, Virginia; VCU, Richmond, Virginia

## Abstract

**Background:**

The 2023 Joint Commission Elements of Performance (EPs) for Antibiotic Stewardship (ASP) document includes a new requirement to implement preauthorization for antimicrobial use in hospitals. At VCUHS, a preauthorization process for restricted antimicrobials has existed since 2000. Prescribers must obtain formal approval for dose and duration of antimicrobials from an ASP or Infectious Diseases (ID) team member to allow inpatient pharmacy to dispense the drug. We employed a stealth stewardship strategy to assess compliance to the antimicrobial preauthorization process and identified opportunities for improvement.

**Methods:**

Pharmacy orders for adult hospitalized patients who received at least one dose of select restricted antimicrobials between August 1, 2022 and October 31, 2022 were reviewed for a documented formal approval in the electronic medical record (EMR). Orders were excluded if not subject to antimicrobial restriction; placed at VCUHS satellite campuses or during de-restricted overnight hours. Stealth use of antimicrobials was defined as the use of a restricted agent without a formal ASP or ID approval.

**Results:**

A total of 179 restricted antimicrobial orders were reviewed during the study period. Of those, 43 (24%) lacked documented approval in the EMR. Of these orders, 20/43 (46.5%) were for patients seen by the ID service, 16/43 (37.2%) were restricted agents continued beyond the initial approval duration, 5/43 (11.6%) were prophylactic antifungal agents approved during prior admissions, and 2/43 (4.7%) were home antimicrobial agents (Fig 1).

**Figure:**
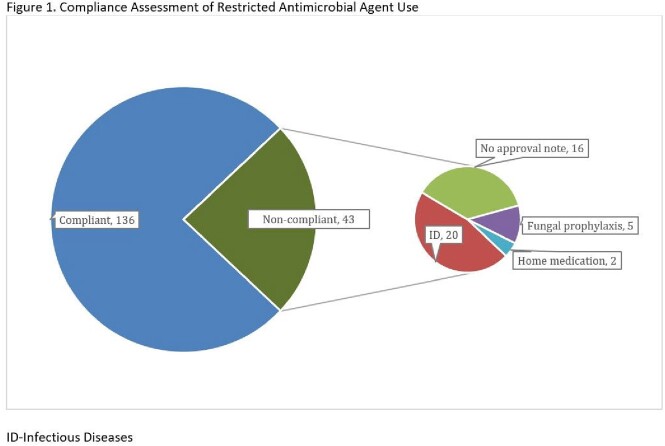

**Conclusion:**

With a stealth stewardship review of restricted antimicrobials, we identified gaps in our stewardship process. Surprisingly, 46% of undocumented restricted antimicrobial use was under the guidance of the ID service. High staff turnover rates, frequent pharmacy and prescriber hand-offs, and lack of persistent education on the restriction process are probable drivers of noncompliance. These breaches in our approval process highlight opportunities for heightened education and feedback. We encourage other institutions to consider similar strategies to evaluate their established processes and ensure compliance with the current Joint Commission requirements.

**Disclosures:**

**All Authors**: No reported disclosures

